# Investigating the Clinical Value in Relation to Implementation and Use of an AI-Generated Fracture Algorithm Tool to Support Clinical Decision-Making

**DOI:** 10.3390/diagnostics16101523

**Published:** 2026-05-18

**Authors:** Mie Strandby Jul, Malene Dybdahl, Janni Jensen, Malene Roland Vils Pedersen, Jane Stigaard, Helle Precht, Ane Simony

**Affiliations:** 1 Department of Ortopaedic and Traumatology, University Hospitals of Southern Denmark, Sygehusvej, 6000 Kolding, Denmark; 2 Department of Radiology, University Hospitals of Southern Denmark, Sygehusvej, 6000 Kolding, Denmark; 3 Department of Radiology, University Hospitals of Southern Denmark, Beriderbakken 4, 7100 Vejle, Denmark; 4 Research and Innovation Unit of Radiology, University of Southern Denmark, 5230 Odense, Denmark; 5 Department of Radiology, Odense University Hospital, 5000 Odense, Denmark; 6 Centre for Clinical Artificial Intelligence (CAI-X), Odense University Hospital, University of Southern Denmark, 5000 Odense, Denmark; 7 Department of Regional Health Research, Faculty of Health, University of Southern Denmark, Campusvej 55, 5000 Odense, Denmark; 8 Discipline of Medical Imaging and Radiation Therapy, School of Medicine, University College Cork, T12 AK54 Cork, Ireland; 9 Radiography Education, VIA University College, 7400 Herning, Denmark; 10 Health Sciences Research Centre, UCL University College, 5230 Odense, Denmark

**Keywords:** artificial intelligence (AI), fracture detection, emergency department, trauma imaging, diagnostic support, clinical implementation, clinician trust

## Abstract

**Background/Objectives:** The use of artificial intelligence (AI) in imaging departments is increasing in Europe. This study assesses the clinical value of an AI fracture algorithm by assessing ease of use, clinicians’ trust, and perceived barriers and benefits of this decision support tool in daily practice across two emergency departments (EDs) in Denmark. **Methods:** An online survey was distributed over four weeks (June–July 2025) to healthcare professionals interpreting radiographs in the ED at Lillebaelt Hospital. The survey included open-ended, closed-ended, and free-text questions addressing AI use. Additionally, an observational study was conducted, including workflow observations and time tracking of patient progression through the ED. Historical injury conference records from February 2023 to 2025 were reviewed to assess changes in patient management before and after AI implementation. **Results:** A total of 56 responses were obtained (24 male, 32 female). Most respondents reported a positive attitude toward the algorithm. Ease of use was rated satisfactory by 51 out of 56 participants, and 48 were satisfied with AI as a clinical decision support tool. Overall trust was high, with more than two thirds (*n* = 38) “agreeing” or “strongly agreeing” that the algorithm reliably detects fractures. However, an asymmetry in clinical trust was observed, whereby clinicians expressed greater confidence in their own assessments when the algorithm indicated the presence of a fracture than when it did not. Value stream analyses showed a delay of 6–23 min between radiograph acquisition and availability of the AI report. No differences were observed in the number of patients with treatment changes before, during, or after full implementation of the algorithm. **Conclusions:** In our limited study population, the AI fracture detection tool was overall well received by clinicians, although some observations indicate that implementation and workflow integration still require improvement. Larger studies are needed to validate the reported barriers and benefits of the AI fracture detection tool.

## 1. Introduction

Suspected fractures after minor trauma are among the most common reasons for presenting to the emergency department (ED) [1]. Radiographs are performed on 35% of patients attending the ED, predominantly orthopedic in origin [2,3]. Delays often occur when less experienced clinicians wait for support from senior colleagues to interpret images. During nights and weekend hours, increased patient volumes and reduced staffing further prolong waiting times. Less commonly, misinterpretation leads to missed fractures, delayed treatment, and additional hospital visits. Accurate, timely diagnosis is therefore essential [4]. To ensure correct diagnosis and treatment, a review of all ED radiographs the following day by a radiologist or reporting radiographer and an orthopedic surgeon at a so-called injury conference (IC) is standard practice. Patients with initial misdiagnoses are contacted for follow-up. This process is time-consuming and utilizes valuable resources.

Artificial intelligence (AI) is increasingly used in medicine, and, according to the newest inventory of AI algorithms approved by the U.S Food and drug Administration (FDA), 76% of those algorithms operate within radiology, with many for fracture detection [5]. These tools can improve diagnostic accuracy and ensure timely treatment. Even though high speed and high accuracy in AI tools have been proven by several studies, the significance of contributed clinical value, as well as the experience with integration into clinical routines, remains limited [4,6,7,8]. The presence of well-validated fracture algorithms alone does not guarantee successful implementation. Challenges such as clinician trust, user acceptance, and integration into clinical workflows and diverse digital systems may still impede AI adoption [9].

Among the most common barriers to AI implementation into radiology practice is radiologists’ lack of acceptance and trust in AI and perceived threats to the professional autonomy of radiologists [10]. While research on implementation often focuses on tool development and data security, barriers from the perspective of healthcare professionals adapting existing AI systems remain unexplored [8,9].

A qualitative multiple case study report identified uncertainty about added clinical value as a key hindrance, lowering acceptance and complicating funding for AI adoption [9]. Demonstrating tangible clinical benefits beyond accuracy and precision is essential to increase clinician acceptance and integrate AI effectively into everyday practice and workflow.

This study aimed to measure the value of an AI fracture algorithm in terms of clinical decision-making, with the following objectives: (1) investigating the clinicians’ satisfaction with the ease of use, (2) measuring clinicians’ trust in an AI-generated diagnosis, (3) identifying key barriers and benefits experienced by staff using the algorithm in daily practice, and finally (4) determining whether the algorithm affected delays or changes in treatment before and after the IC.

## 2. Materials and Methods

**Figure 1:**
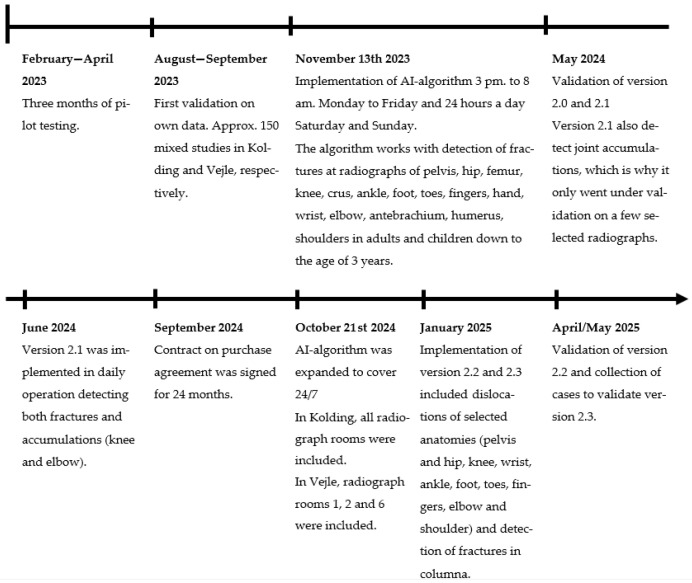
Timeline for implementation of AI fracture detector algorithm at Lillebelt Hospital.

This study was undertaken at Lillebaelt Hospital, University Hospitals of Southern Denmark, which consists of two different geographical locations in Kolding and Vejle. The radiology department has a PACS system (Philips Radiology Information System 11, Eindhoven, The Netherlands) and has an AI fracture detection system installed in the ED, named RB Fracture version 2.4 (Radiobotics, Copenhagen, Denmark). Radiobotics is an AI-driven image analysis algorithm that automatically detects anatomical structures, extracts quantitative measurements, and identifies musculoskeletal abnormalities on radiographs. It operates by applying deep-learning models trained on large annotated datasets to segment relevant regions, analyze morphological patterns, and generate standardized outputs that support clinical decision-making. The fracture detecting algorithm is integrated into the PACS. During use, the clinician opens the radiographic images in PACS as usual and scrolls to the final page, where the radiograph(s) are duplicated and marked with “Radiobotics” in the upper corner. If the algorithm indicates the presence of a fracture, a red box is displayed around the location of the fracture. It is not possible to ascertain whether the algorithm was used in each individual clinical assessment. The AI fracture algorithm was gradually implemented over a two-year period from piloting and validation to full implementation with 24/7 availability of an AI-generated fracture diagnosis [11]. The availability expanded as validation and ongoing improvements were made (Figure 1).

In this context, the terminology of “diagnosing fractures” is defined by the final diagnosis established by a radiologist or the reporting radiographer. In the radiology departments at both locations (Kolding and Vejle), radiographers with additional training in musculoskeletal image interpretation (reporting radiographers) are primarily responsible for conducting the IC. Consequently, the preliminary image evaluation (PIE) is performed exclusively by ED staff, including physicians with varying levels of experience, medical students, specialized trauma nurses, and chiropractic personnel, and places substantial emphasis on the patient’s clinical presentation. During evening and weekend on-call hours, only a single radiologist is available, whose responsibilities are limited to reporting urgent findings that cannot be deferred until the following day. Consequently, it is not feasible to obtain a rapid assessment from either a reporting radiographer or a radiologist for radiographs that can await later evaluation. The AI-based fracture detection algorithm represents an investment by the radiology departments aimed at providing Eds with a decision support tool intended to facilitate a faster and more accurate initial assessment, without necessitating increased on-call radiology staffing or additional training of reporting radiographers.

This project has been approved by the University of Southern Denmark Research Ethics Committee (25/23675) on 4 June 2025 and by the Danish Data Protection Agency (ID 25/27461) on 2 June 2025. All data were fully anonymized.

### 2.1. Survey

The survey was developed in Survey Exact (Xact by Rambøll, Aarhus, Denmark) and consisted of 11 questions relevant for the clinical users regarding the project aim with usage and user satisfaction of the AI-generated fracture algorithm. Questions were designed based on previous surveys used for evaluation [12,13]. The final survey went through pilot testing among five clinicians before it was released for responses. Clinicians who were part of the pilot study were not included in the final dataset. Furthermore, background data on profession, gender, and frequency and years of experience in assessing radiographs were collected. Information about the survey was distributed through flyers in the ED, posters in the orthopedic conference room, daily briefings at the start of each shift, mailing lists to employees, and word of mouth. The survey could be accessed via a link or a QR code and anonymously answered within 30 days. Informed consent was mandatory and was obtained as the first question. If informed consent was not obtained, the survey ended.

### 2.2. Participants

All healthcare professionals in the ED who perform PIE were invited to participate in the survey. Staff in the ED include doctors with different levels of experience, medical students, specialized trauma nurses and chiropractic staff. Junior doctor refers to a foundation doctor, senior doctor refers to a specialty registrar, and non-specialist medical position refers to doctors not in a formal specialty training post. This included clinicians in the Eds in both Kolding and Vejle. Given the limited number of potential respondents among chiropractic and chiropractic students, these two groups were combined to preserve anonymity.

### 2.3. Observational Study

Concurrently, data were extracted from practical observations to investigate whether quantitative observations would support the survey response and whether the barriers reported by the clinicians could be substantiated. Practical observations on clinical workflows were made, including observations of the time each patient spend in each section of the ED (examination, radiography, results and treatment). An author (JS) made value stream analyses [14] by following the workflow of a specialized trauma nurse, both involving daytime and shift/evening time, from when the patient was reported in to when the patient visit ended. Time observations were noted in a predefined diagram at every step for the individual patient, and the exact time was noted. This was conducted for 52 individual patients distributed over five days of observations [15]. Furthermore, we extracted data on the time interval between the transmission of images to PACS and the appearance of AI-generated images with a suggested diagnosis in PACS. Changes in patient treatment were explored based on historical ICs. These conferences are conducted daily (Monday to Friday) and comprise a systematic evaluation of all radiographs and associated clinical documentation from the ED. The assessments are performed by an orthopedic doctor and a reporting radiographer or a radiologist, with the objective of identifying diagnostic discrepancies and potential inadequacies in the radiographic diagnosis and treatment. Two authors (JS and HP) went through daily patient lists from the ICs within two time points (February 2023, before AI fracture algorithm implementation; February 2024, with algorithm assistance from 3 pm to 8 am; and February 2025, with 24/7 algorithm assistance). It was also noted if a phone call was made to the patient after the patient left the hospital. However, the actual information given to the patient during the phone call was not available.

### 2.4. Analysis

The survey answers were analyzed as categorical data using descriptive statistics with frequency analysis and visual representation. Data were described as frequencies (*n*), and observational findings were compared by percentages (%). Differences between the number of patients evaluated at ICs were assessed using the χ^2^-test. Data were analyzed using Excel (version 16.107.2, Microsoft Corporation © 2026, Washington. DC, USA). Two-sided *p*-values < 0.05 were considered statistically significant.

## 3. Results

### 3.1. Survey Participants

**Figure 2:**
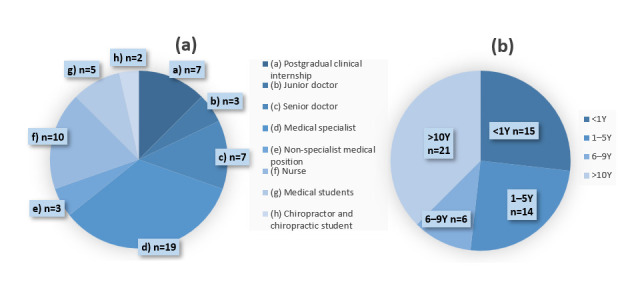
(**a**) Distribution of survey respondents according to postgraduate clinical profession. (**b**) Distribution of survey respondents according to level of experience in assessing radiographs.

Within the two Eds, a total of 56 clinicians participated in the survey. According to institutional records, the maximum possible number of responses was 86 (Appendix A). Among the 56 participants, 32 were female and 24 were male. There were no dropouts. Respondents included medical doctors at different levels, nurses, medical students and chiropractor students with clinical experience (Figure 2a). Most respondents (*n* = 21) had >10 years of experience in assessing radiographs (Figure 2b). In second place (*n* = 15) were respondents with less than one year of experience. Other respondents were divided into groups of 14 and six respondents with 1–5 and 6–9 years of experience, respectively.

Only three respondents assessed radiographs monthly, compared with 15 who undertook tasks weekly and 38 who performed it daily. Regarding the use of the fracture detection algorithm, 17 out of 56 respondents reported using it daily, whereas 22 reported using it weekly.

### 3.2. Clinician’s Satisfaction with AI Algorithm

Overall, most respondents expressed a positive attitude toward the algorithm. A total of 51 out of the total of 56 respondents were satisfied with its ease of use, and 48 reported being satisfied with it as a clinical decision support tool. Furthermore, 46 respondents stated that they preferred having access to the algorithm compared to not having it, while only five indicated they would prefer to work without it. Notably, 45 respondents said they would recommend the tool to colleagues.

### 3.3. Clinicians’ Trust in an AI Diagnosis

An overall trust in the algorithm was reported, with more than two thirds of the participants (*n* = 38) reporting that they either “agree” or “strongly agree” that they trust the algorithm to detect fractures correctly. Eight respondents answered “other” and elaborated with consistent comments that confirm the trust in the algorithm as a support tool, which, however, cannot stand alone without comparing the algorithm’s assessment with the clinical assessment.

Another key finding is the asymmetry in clinical confidence: while 54 out of 56 respondents “agreed” or “strongly agreed” that the algorithm increased their confidence when a fracture was detected, only 32 felt more confident in ruling out a fracture when the algorithm did not detect one, and 21 respondents “disagreed” or “strongly disagreed” with that statement.

### 3.4. Barriers and Benefits with an AI Tool

When respondents reported using the algorithm, their motivations were primarily related to increased diagnostic confidence. Many stated that the tool served as a helpful reminder to consider findings that might otherwise be overlooked, or that it reinforced their own assessments. Curiosity about whether the algorithm would detect additional findings was also a common reason for use. Very few indicated that saving time was a key factor in their decision to use the tool. Less than half of the respondents (*n* = 24) agreed that they spent less time detecting and diagnosing fractures compared with traditional approaches (e.g., clinical decision-making, supervision from colleagues, or radiology reports). Conversely, among those who did not use the algorithm in certain situations, the most frequently cited reasons were delays in receiving the AI result (*n* = 21), a lack of trust in the output (*n* = 14) or a strong personal confidence in the diagnosis that made them feel the tool was unnecessary (*n* = 5).

### 3.5. Observational Study Findings

During the first 12-month period, the AI fracture algorithm was deployed exclusively during weekends and nighttime hours, resulting in an average of 2120 radiographs analyzed per month, with a fracture detection rate of 31.2%. After full-time implementation, the algorithm analyzed an average of 7533 radiographs monthly, with a fracture detection rate of 23.3%. The value stream analyses showed a delay in AI report availability: from when the radiographs were sent to PACS until they appeared with a suggested fracture diagnosis from the AI software (version 2.1.1), a delay was measured with a mean of 8 min (range 6 to 23 min) and a median of 7 min. The variability in delay was investigated further, and practical observations showed that if a radiograph is open in PACS, the fracture algorithm did not update automatically, meaning that the user should close the image and open the same image again before the annotation can become visible. This non-automatic update is suspected to have a major influence on what constituted as a delay in AI report availability. This has been discussed with the manufacturer and was determined to be an issue with the PACS system only. Unfortunately, we never found the reason for this, and there was no correlation with either workload, system or radiograph.

Changes in patient treatment showed no obvious differences between the years before, during and after full implementation of the fracture algorithm (Table 1); no statistically significant differences were observed (χ^2^-test, *p* = 0.71).

## 4. Discussion

The implementation of AI systems in addition to traditional radiological methods offers the potential to enhance the speed and efficiency of diagnostic processes while reducing workload by transferring time-consuming tasks from radiologists to automated systems. A review by Meena et al. claimed that AI performs exceptionally well in academic research settings, and it even surpasses human accuracy in the detection and classification of fractures on radiographs [4]. According to the meta-analysis by Nowroozzi [6], radiologists demonstrated overall superior diagnostic performance in fracture detection on radiographs compared to AI alone. A more nuanced discussion is required to determine why AI-based tools continue to be regarded primarily as assistive instruments and are not yet entrusted with establishing the definitive diagnosis. Although these tools demonstrate strong performance in terms of both precision and accuracy, it is problematic that the body of research examining the clinical outcomes associated with their use remains limited. Similarly, there is a striking lack of shared experience regarding the implementation processes of these AI-based tools.

### 4.1. Survey Study

It was not possible to retrospectively determine whether the level of professional experience influenced trust in the algorithm or barriers to its use. However, 38 respondents reported trusting the algorithm, while an additional eight respondents neither explicitly agreed nor disagreed but noted the importance of interpreting radiographic findings within the broader clinical context. In total, 46 out of 56 respondents expressed either trust or conditional acceptance of the algorithm’s recommendations. Notably, the group of respondents with more than 10 years of experience in radiographic evaluation (*n* = 21) exceeded the number of respondents who expressed neutrality or distrust, indicating that even highly experienced professionals may place trust in AI-supported fracture assessment. At the same time, this trust appears to be nuanced. As highlighted by Gotta et al. [16], a key barrier to full adoption of AI in radiology is the so-called “black box” nature of many algorithms, where clinicians lack insight into how conclusions are derived. This opacity, combined with uncertainty regarding the underlying decision-making processes, may contribute to a more cautious or conditional trust among clinicians, even when they recognize the potential benefits of such tools. It could therefore be considered whether highly experienced professionals, who may have fewer colleagues available for consultation, are more inclined to rely on decision support tools despite these transparency-related concerns.

Overall, the algorithm appears to be used as a supplementary “checklist” to support clinicians’ own assessments, with the proviso that its output must be consistent with the patient’s clinical presentation. A clear preponderance of the responders agreed that if the algorithm suggested a fracture, then a fracture was present. There was less belief that the algorithm was right if it did not show a fracture and the clinician expected a fracture to be present. This finding is not only surprising but also contradictory to what one would expect based on the stated intentions behind fracture detection algorithms. According to the developers, such algorithms are typically designed to prioritize sensitivity over specificity, meaning they are intentionally biased toward identifying more false positives rather than risking false negatives [17].

### 4.2. Observational Study

An average delay of approximately eight minutes, or the requirement for clinicians to re-open the PACS system to access the result of the algorithm, may represent a significant challenge for staff working in the ED. This compromises the availability of AI-based assistance, as clinical decisions may already have been made and senior colleagues consulted before the AI-generated diagnosis becomes accessible. Patients with fractures of the upper limbs are often seen in a fast-track manner to reduce waiting time in the ED. The delay in AI report availability has important clinical significance in a fast-track ED setting and can have a major impact on cases in which the tool is used. Furthermore, the expected time at which the AI diagnosis becomes available cannot be reliably predicted, as the interval required for images to be integrated into PACS varies widely (ranging from 6 to 23 min). Therefore, delay can reduce the use of the AI tool, and more experienced staff might believe more in the clinical assessment than the use of virtual intelligence. A study by Strohm et al. reports that the most dominant hindering factor for a successful implementation was “inconsistent technical performance of AI applications” [9]. The fact that users are required to close and reopen the image constitutes both a technical limitation that must be considered during the implementation process of similar AI-based clinical decision support tools and a significant real-world barrier that would plausibly reduce AI utilization, particularly for experienced staff who open images immediately on acquisition. To draw more conclusions regarding other factors that may be associated with variation in availability such as workload, radiograph-type, etc., additional data are required.

A reduction in potential change in patient treatment was not detected in this study. Earlier studies have shown an improvement in clinicians’ image reading, since misinterpretation rates after AI tool introduction were reduced by 47% [18]. During this study, no significant reduction was observed in the number of patients who were contacted the day after their initial visit. We could not determine if the rate of undetected fractures had been reduced in the study period since we are not able to report the reasoning for contacting the patient, which is a significant limitation to our study. The quality of care does not solely rely on the fracture detection, but also on the treatment chosen, which is part of the reason for the evaluation by a senior consultant the following day.

In addition, we were unable to determine whether the requirement to reopen images in PACS was associated with non-use of the algorithm. In general, it was impossible to determine whether the algorithm was utilized, since the practical observations did not capture whether the tool was used in clinical decision-making in individual cases, nor was its use tracked in retrospective data, such as the number of patients whose treatment plans were modified following evaluation on IC.

## 5. Conclusions

Our study showed great satisfaction with the ease of use of this particular AI fracture detecting algorithm as a decision support tool among clinicians in the ED. Furthermore, clinicians appear to rely heavily on the accuracy of the AI-generated diagnosis, although it is crucial that the proposed diagnosis is consistent with the clinical examination. The delay in radiographic analyses was surprisingly eight minutes on average and could be the most prominent barrier to clinicians using the fracture algorithm. Finally, the rate of patients who needed a subsequent phone call after their initial visit in the ED was unchanged. Further studies with larger data and a study design that differs by profession and experience level are needed to determine the significance of the barriers and benefits found and to reproduce the observational findings in the study.

### Limitations

This study consists of a satisfaction survey, an observational study and a retrospective evaluation of the patients who were contacted after their initial visit to the ED. The study contains the following limitations, which should be considered when interpreting the results. Our survey was anonymized, which is both a limitation and a strength. Only two thirds of the staff invited participated in the survey, and the survey results are based on answers from only 56 responders, which is considered a weakness of the study.

Radiobotics was implemented gradually, which may have affected its adoption and clinical use, as staff likely needed time to recognize its potential and become comfortable integrating it into their daily workflow. Furthermore, AI usage was not logged at the patient level, which makes it impossible to determine whether the algorithm was consulted in cases where treatment plans were subsequently changed. This is a primary limitation, since this data omission does limit the interpretive value of Table 1.

The observational component of the study was only performed over a short period of time, which might have influenced the results. Since we collected data retrospectively, we were unable to record if a phone call after the visit in the ED was due to missed fractures or due to a change in treatment regimes. Furthermore, orthopedic surgical ICs are conducted in a highly manual manner, and adherence to a formal protocol is inconsistent, leading to incomplete data.

## Figures and Tables

**Table 1:** Number of patients who were reviewed at orthopedic radiology ICs in February, without (2023), with partial (2024) and with full (2025) implementation of the AI fracture algorithm, as well as the proportion of patients with a new treatment plan after conference.

Year	Number of Patients at Injury Conferences	Number of Patients (Percentage) with New Treatment Plan
February 2023	1493	115 (7.7%)
February 2024	1268	106 (8.4%)
February 2025	1282	109 (8.5%)

## Data Availability

The raw data supporting the conclusions of this article will be made available by the authors on request provided it is permitted by local hospital authority and national data protection law.

## Abbreviations

The following abbreviations are used in this manuscript:

AI	Artificial intelligence
ED	Emergency department

## Appendix A. Number of Possible Respondents

**Kolding: **
21 nurses + 30 physicians working in the emergency department.
1 chiropractor + 1 chiropractor student in the emergency department.
**Vejle:**
5 nurses + 2 in training in the emergency department.
26 physicians working in the emergency department.
**Total: ** 86 possible respondents.
